# Higher Dietary Choline and Betaine Intakes Are Associated with Better Body Composition in the Adult Population of Newfoundland, Canada

**DOI:** 10.1371/journal.pone.0155403

**Published:** 2016-05-11

**Authors:** Xiang Gao, Yongbo Wang, Edward Randell, Pardis Pedram, Yanqing Yi, Wayne Gulliver, Guang Sun

**Affiliations:** 1 College of Food Science and Engineering, Ocean University of China, No.5, Yu Shan Road, Qingdao, Shandong Province, China; 2 Faculty of Medicine, Memorial University, 300 Prince Philip Drive, St. John’s, NL, Canada; 3 The Department of Endocrinology, the First Affiliated Hospital of Dalian Medical University, Dalian, Liaoning, China; Nathan Kline Institute and New York University School of Medicine, UNITED STATES

## Abstract

**Background:**

Choline is an essential nutrient and betaine is an osmolyte and methyl donor. Both are important to maintain health including adequate lipid metabolism. Supplementation of dietary choline and betaine increase muscle mass and reduce body fat in animals. However, little data is available regarding the role of dietary choline and betaine on body composition in humans.

**Objective:**

To investigate the association between dietary choline and betaine intakes with body composition in a large population based cross-sectional study.

**Design:**

A total of 3214 subjects from the CODING (Complex Disease in Newfoundland population: Environment and Genetics) study were assessed. Dietary choline and betaine intakes were computed from the Willett Food Frequency questionnaire. Body composition was measured using dual-energy X-ray absorptiometry following a 12-hour fast. Major confounding factors including age, sex, total calorie intake and physical activity level were controlled in all analyses.

**Result:**

Significantly inverse correlations were found between dietary choline and betaine intakes, with all obesity measurements: total percent body fat (%BF), percent trunk fat (%TF), percent android fat (%AF), percent gynoid fat (%GF) and anthropometrics: weight, body mass index, waist circumference, waist-to-hip ratio in both women and men (r range from -0.13 to -0.47 for choline and -0.09 to -0.26 for betaine, p<0.001 for all). Dietary choline intake had stronger association than betaine. Moreover, obese subjects had the lowest dietary choline and betaine intakes, with overweight subjects in the middle, and normal weight subjects consumed the highest dietary choline and betaine (p<0.001). Vice versa, when subjects were ranked according to dietary choline and betaine intakes, subjects with the highest intake of both had the lowest %TF, %AF, %GF, %BF and highest %LM among the groups in both sexes.

**Conclusion:**

Our findings indicate that high dietary choline and betaine intakes are significantly associated with favorable body composition in humans.

## Introduction

Obesity is widely recognized as a chronic disease associated with many serious health problems including type 2 diabetes, cardiovascular disease, hypertension and at least a dozen of cancers [1, 2]. In the past three decades, the prevalence of obesity and overweight has risen substantially and affects over 600 million people globally [3]. Due to the high prevalence of obesity and its associated health consequences, effective weight reduction and maintenance strategies are needed. Excessive weight gain is influenced by a range of factors including genetic predisposition, improper nutrition, individual behaviour, environmental and social factors [4]. The role of nutrients in diet, especially micronutrients in obesity has been long debated [5–8].

Choline and betaine are quaternary ammonium compounds, which are obtained from diet or by de novo synthesis in tissues. Both of them are found in a wide variety of foods. Among the most concentrated sources of dietary choline are eggs, beef, pork, liver, seafood and milk, whereas betaine is mainly obtained from grains, cereal, beets and spinach [9–13]. Choline is recognized as an essential nutrient and plays an important role in neurotransmitter synthesis, cell-membrane signaling, lipid transport in lipoproteins and methyl-group metabolism by serving as a precursor for acetylcholine, sphingolipid and phospholipids head groups, and the methyl donor betaine [14, 15]. Betaine serves as a compatible osmolyte and a methyl donor in many pathways, including the side-chain methylation of the homocysteine sulfhydryl to form methionine [16]. Growing evidence shows that choline and betaine are involved in the pathogenies of various diseases, including fatty liver [17], cardiovascular diseases [18], metabolic syndrome [19], and various cancers [12].

The beneficial effect of choline and betaine on reducing body fat has been demonstrated in animals, including rats [20], pigs [21, 22], chickens [23, 24]. Choline and betaine are widely used as dietary additives to promote growth and to improve carcass characteristics (reducing percentage body fat and increasing relative lean mass) in livestock industry [25, 26]. However, studies on the effect of dietary choline and betaine intakes on body composition and furthermore on the development of obesity in humans are scarce, and the limited results are contradictory as well. A cross sectional study in examining Norwegian middle aged and elderly men and women showed that plasma betaine was inversely associated with body mass index (BMI), body fat percentage and waist circumference (WC), while plasma choline had the opposite effect [19]. Similarly, a recent study found that higher serum concentration of betaine rather than choline was associated with better profiles of body fat and fat distribution in Chinese adults [27]. Few studies have investigated the effect of betaine supplementation on body weight and body composition in small samples. A study consisting of 23 lean males reported that, 11 of whom received a 2.5 g/day betaine supplementary with simultaneous 6-week progressive resistance training program significantly improved their body composition by both increasing lean mass and reducing fat mass compared with placebo treatment subjects [28]. However, another study in 42 obese subjects with 12 weeks of 6 g/day betaine supplementation did not improve body composition [29]. Similarly, no change of body composition was found in a study involving 34 young males after 10 days of 2 g/day betaine supplementation [30].

To the best of our knowledge, there is no previous study examining the effects of dietary choline and betaine intakes on body composition in a large general population. Therefore, the current cross-sectional study was designed to investigate the association of dietary choline and betaine intakes with body composition in the general Newfoundland population after controlling for potential confounding factors such as age, sex, total calorie intake and physical activity level.

## Materials and Methods

### Study population

All participants were from the CODING (Complex Diseases in the Newfoundland population: Environment and Genetics) study [31–35]. Eligibility of participants for the CODING study was based upon the following inclusion criteria: 1) ≥19 years of age; 2) at least a third generation Newfoundlander; and 3) without serious metabolic, cardiovascular, or endocrine diseases. A total of 3214 participants (2295 females and 919 males) were employed. Among them, 160 individuals were excluded for having incomplete data, including: 8 individuals missed weight and height, 38 individuals missed waist and hip circumference, 12 individuals missed the results of body scan, 156 individuals missed the results of Food Frequency Questionnaire (FFQ), and 69 individuals missed physical activity questionnaire. All participants provided written and informed consent and the study received ethics approval from the Health Research Ethics Authority (HREA), Memorial University, St. John’s, Newfoundland, Canada, with Project Identification Code #10.33 (latest date of approval: 21 January 2015).

### Anthropometric and body composition measurements

Anthropometrics, body composition measurements were collected following a 12-hour fast. Trained personnel conducted the anthropometric measurements of each subject using standard procedures. Standing height was measured using a fixed stadiometer (to the nearest 0.1 cm). After urinating to empty their bladders, subjects wore standard hospital gowns for all weight measurements using a platform manual scale balance (Health O Meter, Bridgeview, IL; to the nearest 0.1 kg). BMI (kg/m^2^) was calculated as weight in kilograms divided by height squared in meters. WC was defined as the midway point between the iliac crest and the lower rib, and hip by the maximum circumference over the buttocks below the iliac crest. Waist-to-hip ratio (WHR) was division of WC by hip circumference.

Dual Energy X-Ray Absorptiometry (DXA; Lunar Prodigy; GE Medical Systems, Madison, WI) was used for the measurement of percent trunk fat (%TF), percent android fat (%AF), percent gynoid fat (%GF), total percent body fat (%BF) and total percent lean mass (%LM). The Lunar Prodigy software system determines automatically the regions. Trunk fat region is from the top of the shoulders to the top of the iliac crest, while the android fat region is the top of the second lumbar vertebra to the top of the iliac crest and the gynoid fat region extends down iliac crest twice the height of the android area. The enCORE (Ver 12.2, 2008, GE Medical Systems, Madison, WI) software package was used for DXA data acquisition. Quality assurance was performed on the DXA scanner daily and the typical CV during the study period was 1.3% [33, 34].

### Lifestyle and dietary assessment

Information regarding lifestyles of participants was collected through a self-administered screening questionnaire. The questions were related to demographics (age and gender), disease status, smoking status, alcohol consumption, and medication use. A separate questionnaire was administered to women with relation to their menopausal status. Physical activity patterns were measured using the ARIC Baecke Questionnaire, which consists of a Work Index, Sports Index, and Leisure Time Activity Index [36].

Dietary intake of each participant was assessed using a 124 item semi-quantitative Willett FFQ [37, 38]. The Willett FFQ requires subjects reporting the number of weekly servings consumed of common food items over the past 12 months. NutriBase Clinical Nutrition Manager (version 8.2.0; Cybersoft Inc, Phoenix, AZ) software package was used to convert weekly intakes into mean daily intakes of calorie (kcal/day), choline and betaine (mg/day) for each individual [39, 40]. Dietary choline and betaine intakes per kilogram body weight (mg/kg/day) were calculated as daily intakes of choline and betaine (mg/day) divided by body weight in kilogram.

### Data analysis

All data are presented as mean ± standard deviation (SD). To perform effective statistical analysis, total calorie intake, dietary choline, betaine intakes per person per day, and dietary choline, and betaine intakes per kilogram body weight per day were log-transformed to normalize the data distributions. Differences in anthropometrics, body composition measurements and dietary choline and betaine intakes between females and males were assessed with independent Student’s *t* test.

Statistical interaction between dietary choline, betaine intakes and gender on the main outcomes was tested by analysis of covariance (ANCOVA). Partial correlation analysis controlling for age, total calorie intake, and physical activity level was used to evaluate the correlations of dietary choline, betaine intakes with weight, BMI, WC, WHR, %TF, %AF, %GF, %BF and %LM within female and male groups. To overcome the possible influence of smoking, medication use, drinking and menopausal status, participants were also divided into subgroups according to smoking status (yes or no), medication use (yes or no) and alcohol consumption (yes or no). Women were further divided into pre- or post-menopausal groups according to their menopausal status.

According to the criteria recommended by World Health Organization (WHO) [41], obesity status was categorized based on BMI into normal weight (18.5–24.9 kg/m^2^), overweight (25.0–29.9 kg/m^2^) and obese groups (≥30.0 kg/m^2^). As the number of underweight subjects (BMI<18.5 kg/m^2^) was too small (n = 35, female/male = 33/2) to perform any effective statistical analysis, they were excluded from analyses. Obesity status was also categorized by percent body fat according to age and gender specific criteria recommended by Bray [42]. Subjects less than 20 years old were excluded due to unavailability of criteria for this age group by Bray criteria. Dietary choline, betaine intakes and body composition measurements were compared among adiposity groups by using ANCOVA controlling for age, total calorie intake and physical activity level. In order to further explore the relationship between dietary choline, betaine intakes and obesity phenotypes, participants were divided into tertiles (low, medium, or high) based upon dietary choline or betaine intakes expressed in mg/kg/day. Differences of obesity measurements, such as weight, BMI, WC, WHR, %TF, %AF, %GF, %BF and %LM among the three groups of choline or betaine intakes were assessed using ANCOVA controlling for age, total calorie intake and physical activity level.

All statistical analyses were performed using SPSS 20.0 (SPSS Inc., Chicago, IL). All tests were two sided and a p<0.05 was considered to be statistically significant.

## Results

### Physical parameters and dietary characteristics

Demographic, physical and dietary characteristics of the entire population and different gender groups are presented in Table 1. Female subjects were on average 3.4 years older than male subjects. Weight, BMI, WC, WHR, %LM and physical activity were significantly higher in males than females (p<0.001). %TF, %AF, %GF, and %BF were significantly lower in males as compared to females (p<0.001). In terms of dietary intake, male participants consumed significantly higher daily calorie (kcal/day) and choline intake (mg/day) than females (p<0.001), while there was no significant difference for daily betaine intake (mg/day) between different gender groups. When daily choline and betaine intakes were expressed in per kilogram body weight (mg/kg/day), female participants consumed significantly more betaine than males but there was no significant difference for choline intake.

**Table 1:** Obesity indexes and dietary choline, betaine intakes by gender ^1^.

	Entire cohort	Female	Male	*p*^3^
	(*n* = 3054–3214)^2^	(*n* = 2232–2295)^2^	(*n* = 822–919)^2^	
Age (year)	42.74±13.02	43.72±12.52	40.28±13.90	<0.001
Weight (kg)	74.65±16.64	69.68±14.22	87.07±15.77	<0.001
BMI (kg/m^2^)	26.79±5.08	26.36±5.15	27.86±4.72	<0.001
WC (cm)	92.21±14.23	89.92±13.97	97.96±13.22	<0.001
WHR	0.92±0.07	0.89±0.07	0.98±0.06	<0.001
Trunk fat (%)	36.28±9.96	38.65±9.02	30.34±9.74	<0.001
Android fat (%)	41.35±11.54	43.44±10.83	36.10±11.60	<0.001
Gynoid fat (%)	40.03±10.08	44.57±6.73	28.63±7.85	<0.001
Total body fat (%)	33.93±9.62	37.36±7.89	25.33±8.10	<0.001
Total lean (%)	62.39±9.36	59.02±7.55	70.82±8.04	<0.001
Calorie intake (kcal/day)	1985.08±905.30	1870.80±815.18	2295.39±1053.57	<0.001
Physical activity	8.25±1.56	8.16±1.54	8.50±1.61	<0.001
Choline (mg/day)	313.82±237.82	292.34±212.80	372.12±287.36	<0.001
Choline (mg/kg/day)	4.39±3.50	4.37±3.48	4.45±3.57	0.660
Betaine (mg/day)	110.10±90.15	108.45±87.58	114.61±96.70	0.230
Betaine (mg/kg/day)	1.55±1.32	1.62±1.36	1.37±1.21	<0.001

^*1*^All values are mean ± SDs. BMI, body mass index; WC, waist circumference; WHR, waist-to-hip rate.

^*2*^Sample size range in each study group.

^*3*^Significant differences between female and male groups, based on independence sample *Student's t-test*, Statistical significance was set to p<0.05.

### Correlation between dietary choline, betaine intakes and obesity measurements

Significant interactions between dietary choline, betaine intakes and gender on weight, BMI, WC, WHR, %TF, %AF, %GF, %BF and LM% were found (p<0.05 for all). The correlations between dietary choline, betaine intakes and weight, BMI, WC, WHR, %TF, %AF, %GF, %BF and %LM in different gender groups are presented in Table 2. In both female and male subjects, dietary choline, betaine intakes (mg/kg/day) were negatively correlated with weight, BMI, WC, WHR, %TF, %AF, %GF, %BF and positively correlated with %LM. The correlations remained significant after adjusting for age, total calorie intake, and physical activity level. Dietary choline intake had the most pronounced correlation with body weight among all measures in females (r = -0.47, p<0.001), while with %AF in males (r = -0.35, p<0.001). However, the association between dietary betaine intake and BMI was the most pronounced in females (r = -0.25, p<0.001), while TF% had the most prominent correlation with dietary betaine intake in males (r = -0.26, p<0.001).

**Table 2:** Correlations between dietary choline and betaine intakes (mg/kg/day) with body composition^1^.

Choline intake	Female (n = 2232)			Male (n = 822)		
(mg/kg/day)	r^2^	r’^2^	*p* for r’^3^	r^2^	r’^2^	*p* for r’^3^
Weight (kg)	-0.35	-0.47	<0.001	-0.31	-0.30	<0.001
BMI (kg/m^2^)	-0.34	-0.44	<0.001	-0.36	-0.30	<0.001
WC (cm)	-0.36	-0.45	<0.001	-0.42	-0.31	<0.001
WHR	-0.11	-0.14	<0.001	-0.23	-0.13	<0.001
Trunk fat (%)	-0.35	-0.37	<0.001	-0.46	-0.33	<0.001
Android fat (%)	-0.34	-0.37	<0.001	-0.47	-0.35	<0.001
Gynoid fat (%)	-0.30	-0.31	<0.001	-0.35	-0.29	<0.001
Total body fat (%)	-0.36	-0.38	<0.001	-0.44	-0.33	<0.001
Total lean (%)	0.36	0.38	<0.001	0.44	0.33	<0.001
Betaine intake	Female (n = 2232)			Male (n = 822)		
(mg/kg/day)	r^2^	r’^2^	*p* for r’^3^	r^2^	r’^2^	*p* for r’^3^
Weight (kg)	-0.23	-0.24	<0.001	-0.24	-0.20	<0.001
BMI (kg/m^2^)	-0.24	-0.25	<0.001	-0.28	-0.22	<0.001
WC (cm)	-0.24	-0.24	<0.001	-0.29	-0.21	<0.001
WHR	-0.09	-0.09	<0.001	-0.22	-0.17	<0.001
Trunk fat (%)	-0.26	-0.23	<0.001	-0.35	-0.26	<0.001
Android fat (%)	-0.25	-0.23	<0.001	-0.34	-0.25	<0.001
Gynoid fat (%)	-0.20	-0.16	<0.001	-0.23	-0.15	<0.001
Total body fat (%)	-0.26	-0.23	<0.001	-0.34	-0.24	<0.001
Total lean (%)	0.26	0.22	<0.001	0.34	0.25	<0.001

^*1*^Partial correlations between dietary choline, betaine (mg/kg/day) intakes and obesity related indexes were controlling for age, total calorie intake, physical activity level. BMI, body mass index; WC, waist circumference; WHR, waist-to-hip ratio.

^*2*^r: correlation coefficient; r’: partial correlation coefficient.

^*3*^Statistical significance was set to p<0.05.

### Comparison of dietary choline and betaine intakes among different adiposity groups

Subjects were divided into three adiposity groups by gender: normal weight, overweight and obese groups according to BMI recommended by WHO and according to %BF by Bray criteria.

According to BMI, as shown in Table 3 and S1 Fig, weight, BMI, WC, WHR, %TF, %AF, %GF and %BF were significantly higher, while %LM was significantly lower in obese and overweight subjects than normal weight subjects (p<0.001). Dietary choline and betaine intakes expressed in mg/kg/day were significantly lower as adiposity status increased. Compared to the normal weight female and male groups, dietary choline intake (mg/kg/day) was significantly lower by 21.4%, 35.8%, 27.6%, and 44.2% in female overweight, female obese, male overweight and male obese groups, respectively. In addition, dietary betaine intake (mg/kg/day) was similarly lower by 22.8%, 36.0%, 21.6%, and 46.6% in female overweight, female obese, male overweight and male obese groups, respectively, compared to normal weight female and male groups. However, there is no significant difference among the three groups when dietary choline and betaine intakes were expressed as mg/day.

**Table 3:** Comparison of body composition and dietary choline, betaine intakes in different obesity status defined by BMI ^1^.

		Normal weight^3^	Overweight^3^	Obese^3^	*p*^4^
Female	Number^2^	1021	731	447	
	Age (year)	41.44±13.05	45.39±11.94	46.64±11.02	-^5^
	Calorie intake (kcal/day)	1889.94±790.16	1830.36±753.61	1882.86±914.92	-^5^
	Physical activity	8.51±1.55	8.05±1.44	7.55±1.43	-^5^
	**Choline (mg/day)**	298.85±221.11	281.24±169.26	289.19 ±224.96	0.815
	**Choline (mg/kg/day)**	5.05±3.87	3.97±2.46	3.24±2.41	<0.001
	Decline (To Normal weight)		21.4%	35.8%	
	**Betaine (mg/day)**	112.27±87.19	103.72±84.28	107.34±93.88	0.219
	**Betaine (mg/kg/day)**	1.89±1.50	1.46±1.20	1.21±1.05	<0.001
	Decline (To Normal weight)		22.8%	36.0%	<0.001
Male	Number^2^	241	357	222	
	Age (year)	35.71±15.25	42.07±13.53	43.86±11.71	-^5^
	Calorie intake (kcal/day)	2512.09±1056.22	2212.11±920.05	2106.55±1038.91	-^5^
	Physical activity	9.09±1.48	8.44±1.62	8.14±1.62	-^5^
	**Choline (mg/day)**	422.85±326.87	358.08±267.35	341.49±268.27	0.818
	**Choline (mg /kg/day)**	5.84±4.42	4.23±3.18	3.26±2.47	<0.001
	Decline (To Normal weight)		27.6%	44.2%	
	**Betaine (mg/day)**	126.48±97.24	117.12±103.36	97.26±82.29	0.135
	**Betaine (mg/kg/day)**	1.76±1.37	1.38±1.32	0.94±0.81	<0.001
	Decline (To Normal weight)		21.6%	46.6%	

^*1*^All values are mean ± SDs. BMI, body mass index; WC, waist circumference; WHR, waist-to-hip rate.

^*2*^Sample size of study group.

^*3*^The following subdivision is used as normal-weight (18.5–24.9 kg/m^2^), overweight (25.0–29.9 kg/m^2^), obese class (≥30.0 kg/m^2^) according to criteria of the WHO.

^*4*^Significant differences between intervention groups. Data were assessed with ANCOVA controlling for age, total caloric intake, physical activity level. Statistical significance was set to p<0.05.

^*5*^As controlling factor, their differences were not analyzed.

When subjects were divided by %BF, as shown in Table 4 and S1 Fig, similar significant relationship was found among these three adiposity groups as seen in BMI groups. Compared to normal weight female and male groups, dietary choline intake (mg/kg/day) was significantly lower by 17.9%, 32.9%, 29.3%, and 38.6% in female overweight, female obese, male overweight and male obese groups, respectively. Correspondingly dietary betaine intake (mg/kg/day) was significantly lower by 13.3%, 33.0%, 29.7%, and 41.7% in female overweight, female obese, male overweight and male obese groups, respectively, compared to normal weight groups. Furthermore, betaine intake expressed as mg/day was also significantly lower with the increase of %BF in both female and male subjects (p<0.05) but significant difference was not found for choline.

**Table 4:** Composition of body composition and dietary choline, betaine intakes in different obesity status defined by BF%^1^.

		Normal weight^3^	Overweight^3^	Obese^3^	*p*^4^
Female	Number^2^	669	634	797	
	Age (year)	42.75±12.72	46.09±11.21	45.09±11.54	-^5^
	Calorie intake (kcal/day)	1896.10 ± 795.20	1788.57±727.19	1845.31±801.47	-^5^
	Physical activity	8.63±1.52	8.05±1.42	7.63±1.37	-^5^
	**Choline (mg/day)**	298.74±215.54	276.06±167.50	272.55 ±192.23	0.187
	**Choline (mg /kg/day)**	5.04±3.98	4.14±2.47	3.38±2.25	<0.001
	Decline (To Normal weight)		17.9%	32.9%	
	**Betaine (mg/day)**	112.70 ± 83.91	108.40±84.14	101.03±87.19	0.018
	**Betaine (mg /kg/day)**	1.88 ± 1.41	1.63±1.25	1.26±1.10	<0.001
	Decline (To Normal weight)		13.3%	33.0%	
Male	Number^2^	253	207	296	
	Age (year)	38.26 ± 14.75	45.32±12.63	41.13±11.10	-^5^
	Calorie intake (kcal/day)	2472.08±1076.60	2060.74±809.53	2152.54±1018.52	-^5^
	Physical activity	9.15±1.51	8.34±1.48	7.84±1.46	-^5^
	**Choline (mg/day)**	417.63±328.69	322.11±183.07	322.57±225.43	0.134
	**Choline (mg/kg/day)**	5.42±4.06	3.83±2.22	3.33±2.51	<0.001
	Decline (To Normal weight)		29.3%	38.6%	
	**Betaine (mg/day)**	133.88±107.55	104.36±78.83	98.94±86.13	0.025
	**Betaine (mg/kg/day)**	1.75±1.42	1.23±0.90	1.02±0.90	<0.001
	Decline (To Normal weight)		29.7%	41.7%	

^*1*^All values are mean ± SDs. BMI, body mass index; WC, waist circumference; WHR, waist-to-hip rate.

^*2*^Sample size of study group.

^*3*^Subgroups were created by percent of body fat according to the age and gender specific criteria recommended by Bray.

^*4*^Significant differences between intervention groups. Data were assessed with ANCOVA controlling for age, total calorie intake and physical activity level. Statistical significance was set to p<0.05.

^*5*^As controlling factor, their differences were not analyzed.

### Comparison of body composition in different dietary choline and betaine intakes groups

Subjects were divided into tertiles (low, medium, or high) according to dietary choline and betaine intakes (mg/kg/day). As shown in Tables 5, 6 and S2 Fig, a significant ‘dose-dependent’ declining in weight, BMI, WC, WHR, %TF, %AF, %GF and %BF was found with the increase of dietary choline and betaine intakes in both females and males after controlling for age, total calorie intake, and physical activity. %LM presented a significantly ‘dose dependent’ increasing trend. Compared to low dietary choline intake group, female subjects of high dietary intake group had significantly lower weight, BMI, WC, WHR (-11.85 kg, -4.24 kg/m^2^, -11.79 cm, -0.02), and %TF, %AF, %GF, %BF(-7.59%, -8.89%, -4.65%, -6.75%) but higher %LM (6.30%). Male subjects of high dietary choline intake group had significantly lower weight (-11.19 kg), BMI (-3.87 kg/m^2^), WC (-12.82 cm), WHR (-0.03), %TF (-10.56%), %AF (-12.66%), %GF (-5.94%), %BF (-8.35%), but higher %LM (8.25%) compared with low choline intake group. While, the average differences of obesity measures between high and low dietary betaine intake (mg/kg/day) groups were -7.42 kg (weight), -2.77 kg/m^2^ (BMI), -7.31 cm (WC), -0.02 (WHR), -6.19% (%TF), -6.24% (%AF), -3.13 (%GF), -4.55% (%BF), 4.00% (%LM) in females, and -8.60 kg (weight), -3.01 kg/m^2^ (BMI), -8.81 cm (WC), -0.03 (WHR), -7.77% (%TF), -8.92% (%AF), -4.19% (%GF), -6.19% (%BF), 6.19 (%LM) in males.

**Table 5:** Comparison of obesity indexes according to dietary choline (mg/kg/day) intake^1^.

	Choline (mg/kg/day)	Low^2^	Medium^2^	High^2^	*p*^3^
Female	Number	744	744	744	
	Age (year)	44.56±11.48	44.37±11.95	42.16±13.77	-^4^
	Weight (kg)	75.95±16.39	68.93±12.30	64.10±10.58	<0.001
	BMI (kg/m^2^)	28.59±5.74	26.15±4.67	24.35±4.02	<0.001
	WC (cm)	96.03±15.02	89.49±12.80	84.24±11.31	<0.001
	WHR	0.90±0.07	0.89±0.07	0.88±0.06	<0.001
	Trunk fat (%)	42.31±7.94	38.89±8.32	34.72±9.10	<0.001
	Android fat (%)	47.72±9.38	43.79±10.00	38.83±11.14	<0.001
	Gynoid fat (%)	46.67±6.37	45.01±6.30	42.02±6.69	<0.001
	Total body fat (%)	40.59±7.08	37.61±7.17	33.84±7.87	<0.001
	Total lean (%)	56.00±6.72	58.76±6.89	62.30±7.66	<0.001
	Calorie intake (kcal/day)	1346.45±476.78	1826.96±538.96	2435.87±935.54	-^4^
	Physical activity	7.77±1.46	8.07±1.46	8.64±1.56	-^4^
	Choline (mg/day)	162.82±50.16	251.21±50.02	463.21±288.89	<0.001
	Choline (mg/kg/day)	2.17±0.55	3.66±0.44	7.30±4.69	<0.001
		(0.36~2.94)	(2.95~4.51)	(4.52~51.27)	
	Betaine (mg/day)	68.70±59.41	107.95±75.97	148.46±102.77	<0.001
	Betaine (mg/kg/day)	0.92±0.75	1.579±1.06	2.35±1.67	<0.001
Male	Number	274	274	274	
	Age (year)	45.11±12.61	41.66±12.90	34.81±14.42	-^4^
	Weight (kg)	92.07±16.42	86.87±14.56	80.88±13.30	<0.001
	BMI (kg/m^2^)	29.66±4.89	27.71±4.28	25.79±3.73	<0.001
	WC (cm)	103.49±12.50	97.84±11.65	90.67±11.53	<0.001
	WHR	0.99±0.05	0.98±0.05	0.96±0.06	0.002
	Trunk fat (%)	34.99±7.34	30.80±8.14	24.43±10.31	<0.001
	Android fat (%)	41.81±8.60	36.38±9.64	29.15±12.60	<0.001
	Gynoid fat (%)	31.32±7.14	28.48±6.73	25.38±8.35	<0.001
	Total body fat (%)	29.07±6.67	25.38±6.72	20.72±8.30	<0.001
	Total lean (%)	67.11±7.20	70.74±6.43	75.36±8.12	<0.001
	Calorie intake (kcal/day)	1606.82±553.64	2170.41±616.33	3117.72±1.22	-^4^
	Physical activity	7.90±1.50	8.54±1.44	9.20±1.68	-^4^
	Choline (mg/day)	185.84±54.77	308.54±64.53	622.54±374.07	<0.001
	Choline (mg/kg/day)	2.04±0.53	3.56±0.48	7.73±4.51	<0.001
		(0.51~2.77)	(2.77~4.49)	(4.49~35.90)	
	Betaine (mg/day)	73.98±63.15	110.24±73.23	160.29±122.86	<0.001
	Betaine (mg/kg/day)	0.82±0.72	1.29±0.87	2.01±1.56	<0.001

^*1*^All values are mean ± SDs. BMI, body mass index; WC, waist circumference; WHR, waist-to-hip ratio.

^*2*^The subjects were divided to low, medium and high choline intake groups based on mg/kg/day.

^*3*^Significant differences between intervention groups. Data were assessed with ANCOVA controlling for age, total caloric intake, physical activity level. Statistical significance was set to p<0.05.

^*4*^As controlling factor, their differences were not analyzed.

**Table 6:** Comparison of obesity indexes according to dietary betaine (mg/kg/day) intake^1^.

	Betaine (mg/kg/day)	Low^2^	Medium^2^	High^2^	*p*^3^
Female	Number	744	744	744	
	Age (year)	44.10±11.75	43.36±12.60	43.61±13.04	-^4^
	Weight (kg)	73.78±15.76	68.72±13.29	66.36±11.92	<0.001
	BMI (kg/m^2^)	27.91±5.56	25.97±4.88	25.14±4.53	<0.001
	WC (cm)	94.08±14.86	88.78±13.28	86.77±12.52	<0.001
	WHR	0.90±0.07	0.89±0.07	0.88±0.06	0.004
	Trunk fat (%)	41.47±8.16	38.14±8.84	36.28±9.24	<0.001
	Android fat (%)	46.90 ± 9.59	42.72±10.70	40.66±11.14	<0.001
	Gynoid fat (%)	46.28±6.25	44.24±6.62	43.15±6.92	<0.001
	Total body fat (%)	39.83±7.21	36.91±7.67	35.28±8.06	<0.001
	Total lean (%)	56.80±6.79	59.42±7.29	60.80±7.66	<0.001
	Calorie intake (kcal/day)	1558.28±714.21	1871.16±815.45	2189.17±789.94	-^4^
	Physical activity	7.85±1.54	8.17±1.47	8.67±l.53	-^4^
	Choline (mg/d)	230.22±177.08	294.10±221.97	353.87±218.41	<0.001
	Choline (mg/kg/day)	3.27±2.99	4.38±3.32	5.49±3.73	<0.001
	Betaine (mg/d)	37.13±18.49	86.93±23.64	201.37±89.07	<0.001
	Betaine (mg/kg/day)	0.51±0.23	1.27±0.25	3.08±1.39	<0.001
		(0.03~0.88)	(0.89~1.77)	(1.78~12.73).	
Male	Number	274	274	274	
	Age (year)	43.15±13.40	39.47±13.76	39.02±14.49	-^4^
	Weight (kg)	90.95±17.09	86.61±14.34	82.35±13.71	<0.001
	BMI (kg/m^2^)	29.19±4.89	27.82±4.36	26.18±4.05	<0.001
	WC (cm)	101.54±13.45	97.78±11.71	92.73±12.32	<0.001
	WHR	0.99±0.06	0.98±0.05	0.96±0.06	<0.001
	Trunk fat (%)	33.51±8.82	31.08±8.11	25.74±10.40	<0.001
	Android fat (%)	39.69±10.28	36.98±9,81	30.77±12.76	<0.001
	Gynoid fat (%)	29.98±7.58	29.48±6.79	25.79±8.32	<0.001
	Total body fat (%)	27.79±7.72	25.86±6.78	21.60±8.32	<0.001
	Total lean (%)	68.30±8.15	70.36±6.50	74.49±8.06	<0.001
	Calorie intake (kcal/day)	1867.65±860.72	2242.66±925.86	2724.76±1.03	-^4^
	Physical activity	8.17±1.61	8.39±1.54	9.05±1.60	-^4^
	Choline (mg/day)	282.73±218.72	352.91±234.20	466.45±324.37	<0.001
	Choline (mg/kg/day)	3.19±2.45	4.15±2.79	5.77±4.10	<0.001
	Betaine (mg/day)	38.74±19.13	88.10±20.28	217.06±101.67	<0.001
	Betaine (mg/kg/day)	0.43±0.19	1.02±0.19	2.67±1.28	<0.001
		(0.03~0.73)	(0.74~1.43)	(1.44~10.99)	

^*1*^All values are mean ± SDs. BMI, body mass index; WC, waist circumference; WHR, waist-to-hip ratio.

^*2*^The subjects were divided to low, medium and high choline intake groups based on mg/kg/day.

^*3*^Significant differences between intervention groups. Data were assessed with ANCOVA controlling for age, total caloric intake, physical activity level. Statistical significance was set to p<0.05.

^*4*^As controlling factor, their differences were not analyzed.

## Discussion

To the best of our knowledge, this is the first large cross-sectional study specifically designed to investigate the association between dietary choline, betaine intakes and DXA-derived body composition. We found significant associations of higher dietary choline and betaine intakes with better body composition profile (lower %BF, higher %LM). Furthermore, the inverse correlation of dietary choline intake with obesity measures was stronger than that of betaine.

Association study is relatively easy to do but hard to do well, especially in large population based study. The first challenge is the accurate measurement of physiological and biological markers because sophisticated equipment is required, and long period of time is needed to recruit large number of subjects. In the present study, body composition in all subjects was measured using the same DXA system by certified technicians over 12 years. DXA produces accurate measurement of adipose tissue in the body with a low margin of error and is considered to be one of the most reliable measurements of adiposity [33]. The second challenge lies in the identification and measurements of numerous confounding factors that can potentially affect the relationship of the major factors under investigation. In most studies, unfortunately, information on these confounding factors is largely unavailable [43, 44]. There is no doubt that age is a primary important factor that affects the development of obesity [45]. Total dietary calorie intake is a critical factor in maintaining energy balance [46]. In addition, physical activity level is likely the most important non-dietary factor that can influence energy consumed, amount of choline and betaine intakes and ultimately body composition [46, 47]. In the present study, all of these confounding factors have been properly adjusted. The beneficial effect of dietary choline and betaine intakes on obesity was demonstrated in every obesity measurement in both women and men independent of these factors. Other confounding factors, menopause, smoking, medication and alcohol consumption were taken into consideration as well. Menopause is an important factor related to changes in body composition as menopause is usually accompanied by dramatic changes in sex hormones that can predispose women to weight gain [48]. We found that the associations between dietary choline, betaine intakes and body composition are equally profound in both pre- and post-menopausal women (S1 Table). Smoking status, medication use and alcohol consumption are potentially important covariates known to affect appetite and body weight regulation [49–51]. After separating subjects according to these covariates, the beneficial association remained significant (S2–S4 Tables).

We used the unit of mg/kg/day to express choline and betaine intakes in all analyses because dietary intake of both is highly correlated with body weight. It would be physiologically inappropriate not to account for body mass when comparing dietary intake among subjects with large difference of body weight. Physiological dynamics of nutritive intake is in many ways similar to that of pharmacodynamics where in the case of choline and betaine, the same dose may be adequate for a person with 60 kg body weight, but insufficient for a person with a body weight of 100 kg or more. The use of mg/kg/day in the present study eliminates the bias of body weight variation in the population and reveals the true relationship between dietary choline, betaine intakes and body composition. Otherwise, we also analyzed the data for mg/day and the negative associations were also significant but not strong (S5 Table). Furthermore, same findings were obtained when obesity status was classified according to either BMI or %BF by Bray criteria. Obese subjects had the lowest dietary choline and betaine intakes. Vice versa, subjects with the highest dietary choline or betaine intakes had the lowest obesity measurements. And the associations were stronger with dietary choline than dietary betaine. Moreover, the associations seem different among fat distribution. The associations were stronger between dietary choline and betaine intakes with %TF, %AF than %GF. Similar findings were reported on the association of serum rather than dietary betaine with body fat percentage and fat distribution [19, 27]. However, an opposite relationship between plasma choline and obesity measurements was reported [19], while chen *et al* found a negative association between serum choline and body weight and BMI [27]. The relationship between dietary intakes and serum levels of choline and betaine is not clear. A small study consisting of children with autism reported a positive correlation between dietary intakes and plasma levels of the nutrients [52]. It is reported that the serum choline level is lower than betaine, although dietary choline intake is higher than betaine in the general populations [12, 19, 27, 53]. This difference between dietary intakes and serum levels highlights the importance to perform independent studies and suggesting that relationship between dietary choline and betaine with obesity might differ from that with serum levels.

To date, a few small interventional studies have examined the effect of betaine supplementation on body fat but no study is available on the effect of choline supplementation in humans. A significant decrease in fat mass and percent body fat was observed among 11 male participants received a periodical training program [28]. However, the positive effect may have been due to the greater physical activity levels of participants. Two other studies failed to replicate the results [29, 30]. Animal studies have provided the main evidence of the beneficial effect of dietary choline and betaine on body fat and muscle. Choline or betaine supplementation on the growth performance and carcass characteristics of gilts, other pigs, meat ducks, chickens and mice were well examined [20–26]. These studies have generally indicated that choline and betaine supplementation can promote growth and reduce fat deposition in various animals. Our findings have filled the knowledge gap of the relationship between dietary choline, betaine intakes and body composition in humans. The effect of dietary choline might be more complex. Jacobs *et al* found that a choline-deficient diet decreased fat mass in mice with high fat diet or genetic defect induced obesity [54, 55]. Another study showed that high fat feeding led to weight gain in choline-replete mice, whereas choline deficiency did not affect body or adipose depot weights [56]. However, these animal experiments were performed under extreme condition: choline depletion which virtually does not exist in general human population.

The exact mechanisms by which choline and betaine improve body composition are unclear, while several mechanisms have been suggested. It has been postulated that choline and betaine supplementation could promote fatty acid β-oxidation in pigs and humans by enhancing muscle carnitine accretion and thereby increase carnitine palmitoyl transferase I-mediated free fat acid translocation into the mitochondria [57–61]. Choline can also be metabolized to betaine and plays a role in altering body composition. The betaine-related methylation of homocysteine to methionine may reduce the availability of substrate (acetyl-coenzyme A) for fatty acid synthesis [60]. Betaine supplementation could decrease the capacity for fatty acid and triglyceride synthesis by decreasing the activity of acetyl-CoA carboxylase, fatty acid synthase and malic enzyme and their mRNA expression in abdominal adipose tissue [61, 62]. Betaine may also reduce uptake of triglycerides from circulating lipoproteins by decreasing the mRNA expression of lipoprotein lipase [62]. Dietary betaine in pigs mobilized fat degradation in adipose tissue by increasing hormone sensitive lipase activity [63]. Betaine supplementation may promote protein synthesis by stimulating growth hormone secretion and improving insulin and IGF-1 receptor signaling [64–66]. Accumulation of betaine in cells resulting in increased sarcoplasmic osmolality may also contribute to the increase in muscle mass [67, 68].

Although our study provides clear evidence on association of increased dietary choline and betaine intakes with favorable body composition, a number of limitations of the work should be considered. Our use of FFQ to evaluate patterns of dietary intake raises the possibility of recall bias by subjects [38]. However, FFQ is the most widely used questionnaire for the assessment of dietary intake at the population level and has been successfully validated and used in a Canadian populations [69–71], including in the Newfoundland population [72]. Thus, we consider the Willett FFQ to be a reasonable method for the assessment of dietary intake patterns in this study. Another potential weakness in the present study is related to its cross-sectional design. Cross sectional study alone does not establish a cause effect relationship. Direct intervention studies or clinical trials with reasonable sample size and well matched subjects between treatment and control groups are warranted. Finally, although multiple factors, including socio-demographic characteristics, lifestyle habits, physical activities and energy intake were comprehensively adjusted in our analysis, genetic factors, dietary nutrient factors absorbed when consuming choline or betaine-rich foods and unknown or poorly measured factors could not be completely ruled out.

## Conclusions

In conclusion, the present study provides solid evidence for the first time, in the large Newfoundland population, that higher dietary choline and betaine intakes were associated with a more favorable body composition (lower body fat and higher lean body mass) in both women and men. In addition, this favorable association was independent of age, gender, total calorie intake, physical activity level, menopausal status, smoking status, medication use, and alcohol consumption. The beneficial correlation for choline seems better than betaine.

## Supporting Information

S1 Fig Variations of dietary choline, betaine intakes (mg/kg/day) in different obesity status. A. variations of dietary choline intake (mg/kg/day) in different obesity status grouped based on BMI criteria; B. variations of dietary betaine intake (mg/kg/day) in different obesity status grouped based on BMI criteria; C. variations of dietary choline intake (mg/kg/day) in different obesity status grouped based on %BF recommended by Bray; D. variations of dietary betaine intake (mg/kg/day) in different obesity status grouped based on %BF recommended by Bray; **p<0.01 compared with normal weight group, ##p<0.01 compared with overweight group.
(TIF)

S2 Fig Variations of total percent body fat and lean mass among subjects with low, medium and high dietary choline and betaine intakes (mg/kg/day). A. variations of total body fat according to dietary choline intake; B. variations of total lean mass according to dietary choline intake; C. variations of total body fat according to dietary betaine intake; D. variations of total lean mass according to dietary betaine intake. **p<0.01 compared with low dietary choline or betaine intakes; ##p< 0.01 compared with medium dietary choline or betaine intakes.
(TIF)

S1 Table Partial correlations between dietary choline, betaine intakes (mg/kg/day) and body composition variables in females based on menopausal status. (DOC)

S2 Table Partial correlations between dietary choline, betaine intakes (mg/kg/day) and body composition variables for Newfoundland population based on medication use. (DOC)

S3 Table Partial correlations between dietary choline, betaine intakes (mg/kg/day) and body composition variables for Newfoundland population based on smoking status. (DOC)

S4 Table Partial correlations between dietary choline, betaine intakes (mg/kg/day) and body composition variables for Newfoundland population based on alcohol status. (DOC)

S5 Table Correlations between dietary choline and betaine intakes (mg/day) with body composition. (DOC)

## References

[1] Calle EE, Kaaks R. Overweight, obesity and cancer: epidemiological evidence and proposed mechanisms. Nat Rev Cancer. 2004;4: 579–591. 15286738 10.1038/nrc1408

[2] Kopelman PG. Obesity as a medical problem. Nature. 2000;404: 635–643. 10766250 10.1038/35007508

[3] Ng M, Fleming T, Robinson M, Thomson B, Graetz N, Margono C, et al Global, regional, and national prevalence of overweight and obesity in children and adults during 1980–2013: a systematic analysis for the Global Burden of Disease Study 2013. The Lancet. 2014;384: 766–781. 10.1016/S0140-6736(14)60460-8 PMC4624264 24880830

[4] Morris MJ, Beilharz JE, Maniam J, Reichelt AC, Westbrook RF. Why is obesity such a problem in the 21st century? The intersection of palatable food, cues and reward pathways, stress, and cognition. Neurosci Biobehav Rev. 2014 12 10 10.1016/j.neubiorev.2014.12.002 25496905

[5] Schoeller DA, Buchholz AC. Energetics of obesity and weight control: does diet composition matter? J Am Diet Assoc. 2005;105: S24–28. 15867892 10.1016/j.jada.2005.02.025

[6] Hu FB. Diet, nutrition, and obesity Obesity Epidemiology. Oxford University Press: New York 2008: 275–300.

[7] García OP, Long KZ, Rosado JL. Impact of micronutrient deficiencies on obesity. Nutr Rev. 2009; 67: 559–572. 10.1111/j.1753-4887.2009.00228.x 19785688

[8] García OP, Ronquillo D, del Carmen Caamaño M, Martínez G, Camacho M, López V, et al Zinc, iron and vitamins A, C and E are associated with obesity, inflammation, lipid profile and insulin resistance in Mexican school-aged children. Nutrients. 2013;5: 5012–5030. 10.3390/nu5125012 24335710 PMC3875915

[9] Nagata C, Wada K, Tamura T, Konishi K, Kawachi T, Tsuji M, et al Choline and Betaine Intakes Are Not Associated with Cardiovascular Disease Mortality Risk in Japanese Men and Women. The Journal of nutrition. 2015;145: 1787–1792. 10.3945/jn.114.209296 26063062

[10] Vennemann FBC, Ioannidou S, Valsta LM, Dumas C, Marga C, Mensink GBM, et al Dietary intake and food sources of choline in European populations. Brit J Nutr. 2015;114: 2046–2055. 10.1017/S0007114515003700 26423357

[11] Wallace T C, Fulgoni V L III. Assessment of Total Choline Intakes in the United States. J Am Coll Nutr. 2016;35: 108–112. 10.1080/07315724.2015.1080127 26886842

[12] Ueland PM. Choline and betaine in health and disease. J Inherit Metab Dis. 2011;34: 3–15. 10.1007/s10545-010-9088-4 20446114

[13] Zeisel SH, Da Costa KA. Choline: an essential nutrient for public health. Nutrition reviews 2009;67:615–623. 10.1111/j.1753-4887.2009.00246.x 19906248 PMC2782876

[14] Penry J, Manore M. Choline: an important micronutirent for maximal endurance-exercise performance? Int J Sport Nutr Exerc Metab. 2008;18: 191–203. 18458362 10.1123/ijsnem.18.2.191

[15] Leermakers ETM, Moreira EM, Kiefte-de Jong JC, et al Effects of choline on health across the life course: a systematic review. Nutr Rev. 2015;73: 500–522. 10.1093/nutrit/nuv010 26108618

[16] Craig SA. Betaine in human nutrition. Am J Clin Nutr 2004;80:539–549. 15321791 10.1093/ajcn/80.3.539

[17] Buchman AL, Dubin MD, Moukarzel AA, Jenden DJ, Roch M, Rice KM, et al Choline deficiency: a cause of hepatic steatosis during parenteral nutrition that can be reversed with intravenous choline supplementation. Hepatology. 1995;22: 1399–1403. 7590654

[18] Rajaie S, Esmaillzadeh A. Dietary choline and betaine intakes and risk of cardiovascular diseases: review of epidemiological evidence. ARYA atherosclerosis. 2011;7: 78 22577451 PMC3347848

[19] Konstantinova SV, Tell GS, Vollset SE, Nygard O, Bleie Ø, Ueland PM. Divergent associations of plasma choline and betaine with components of metabolic syndrome in middle age and elderly men and women. J Nutr. 2008;138: 914–20. 18424601 10.1093/jn/138.5.914

[20] Hongu N, Sachan DS. Caffeine, carnitine and choline supplementation of rats decreases body fat and serum leptin concentration as does exercise. J Nutr. 2000;130: 152–157. 10720162 10.1093/jn/130.2.152

[21] Siljander-Rasi H, Peuranen S, Tiihonen K. Effect of equimolar dietary betaine and choline addition on performance, carcass quality and physiological parameters of pigs. J Anim Sci, 2003;76: 55–62.

[22] Daily JW, Hongu N, Mynatt RL, Sachan DS. Choline supplementation increases tissue concentrations of carnitine and lowers body fat in guinea pigs. J Nutr Biochem 1998;9: 464–70.

[23] Saunderson CL, Mackinlay J. Changes in body-weight, composition and hepatic enzyme activities in response to dietary methionine, betaine and choline levels in growing chicks. Br J Nutr. 1990;63: 339–49. 1692235 10.1079/bjn19900120

[24] Hassan RA, Attia YA, El-Ganzory EH. Growth, carcass quality and serum constituents of slow growing chicks as affected by betaine addition to diets containing 1. Different levels of choline. Int. J. Poult. Sci. 2005;4: 840–850.

[25] Pollmann DS, Danielson DM, Peo ER. Effects of microbial feed additives on performance of starter and growing-finishing pigs. J Anim Sci. 1980;51: 577–581.

[26] Lever M, Slow S. The clinical significance of betaine, an osmolyte with a key role in methyl group metabolism. Clin Biochem. 2010;43:732–744. 10.1016/j.clinbiochem.2010.03.009 20346934

[27] Chen YM, Liu Y, Liu YH, Wang X, Guan K, Zhu HI. Higher serum concentrations of betaine rather than choline is associated with better profiles of DXA-derived body fat and fat distribution in Chinese adults. Int J Obes. 2015;39: 465–471. 10.1038/ijo.2014.158 25152241

[28] Cholewa JM, Wyszczelska-Rokiel M, Glowacki R, Jakubowski H, Matthews T, Wood R, et al Effects of betaine on body composition, performance, and homocysteine thiolactone. J Int Soc Sports Nutr. 2013;10: 39 10.1186/1550-2783-10-39 23967897 PMC3844502

[29] Schwab U, Torronen A, Toppinen L, Alfthan G, Saarinen M, Aro A, et al Betaine supplementation decreases plasma homocysteine concentrations but does not affect body weight, body composition, or resting energy expenditure in human subjects. Am J Clin Nutr. 2002;76: 961–967. 12399266 10.1093/ajcn/76.5.961

[30] Favero S, Roschel H, Artioli G, Ugrinowitsch C, Tricoli V, Costa A, et al Creatine but not betaine supplementation increases muscle phosphorylcreatine content and strength performance. Amino Acids. 2012;42: 2299–2305. 10.1007/s00726-011-0972-5 21744011

[31] Sun G, French CR, Martin GR, Younghusband B, Green RC, Xie YG, et al Comparison of multifrequency bioelectrical impedance analysis with dual-energy X-ray absorptiometry for assessment of percentage body fat in a large, healthy population. Am J Clin Nutr. 2005;81:74–78. 15640463 10.1093/ajcn/81.1.74

[32] Sun G, Vasdev S, Martin G, Gadag V, Zhang HW. Altered calcium homeostasis is correlated with abnormalities of fasting serum glucose, insulin resistance and β-cell function in the Newfoundland population. Diabetes. 2005;54: 3336–3339. 16249463 10.2337/diabetes.54.11.3336

[33] Kennedy AP, Shea JL, Sun G. Comparison of the classification of obesity by BMI vs. dual-energy X-ray absorptiometry in the newfoundland population. Obesity. 2009;17: 2094–2099. 10.1038/oby.2009.101 19360011

[34] Shea JL, King MT, Yi Y, Gulliver W, Sun G. Body fat percentage is associated with cardiometabolic dysregulation in BMI-defined normal weight subjects. Nutr Metab Cardiovasc Dis. 2012;22:741–747. 10.1016/j.numecd.2010.11.009 21215604

[35] Fontaine-Bisson B, Thorburn J, Gregory A, Zhang H and Sun G. Melanin-concentrating hormone receptor 1 polymorphisms are associated with components of energy balance in the Newfoundland CODING study. Am J Clin Nutr. 2014;99:384–391. 10.3945/ajcn.113.073387 24305679

[36] Baecke JA, Burema J, Frijters JE. A short questionnaire for the measurement of habitual physical activity in epidemiological studies. Am J Clin Nutr. 1982;36:936–942. 7137077 10.1093/ajcn/36.5.936

[37] Willett WC, Sampson L, Stampfer MJ, Rosner B, Bain C, Witschi J, Hennekens CH, Speizer FE. Reproducibility and validity of a semiquantitative food frequency questionnaire. Am J Epidemiol. 1985;122: 51–65. 4014201 10.1093/oxfordjournals.aje.a114086

[38] Subar AF, Thompson FE, Kipnis V, Midthune D, Hurwitz P, McNutt S, et al Comparative validation of the Block, Willett, and National Cancer Institute food frequency questionnaires: the Eating at America’s Table Study. Am J Epidemiol. 2001;154:1089–1099. 11744511 10.1093/aje/154.12.1089

[39] Cahill F, Shahidi M, Shea J, Wadden D, Gulliver W, Randell E, et al High Dietary magnesium intake is associated with low insulin resistance in the Newfoundland population. PLoS One. 2013, 8:e58278 10.1371/journal.pone.0058278 23472169 PMC3589265

[40] Pedram P, Sun G. Hormonal and Dietary Characteristics in Obese Human Subjects with and without Food Addiction. Nutrients. 2014; 7: 223–238. 10.3390/nu7010223 25558907 PMC4303835

[41] WHO. Obesity: preventing and managing the global epidemic. Report of a WHO Consultation. WHO Technical Report Series 894. Geneva: World Health Organization, 2000. 11234459

[42] Bray GA. Contemporary Diagnosis and Management of Obesity and the Metabolic Syndrome. 3rd edn Handbooks in Health Care: Newtown, PA, 2003.

[43] Pocock SJ, Collier TJ, Dandreo KJ, Stavola BL, Goldman MB, Kalish LA, Kasten LE, McCormack VA. Issues in the reporting of epidemiological studies: a survey of recent practice. BmJ, 2004;329: 883 15469946 10.1136/bmj.38250.571088.55 PMC523109

[44] Mann CJ. Observational research methods. Research design II: cohort, cross sectional, and case-control studies. Emergency Medicine Journal. 2003;20: 54–60. 12533370 10.1136/emj.20.1.54 PMC1726024

[45] Jackson AS, Stanforth PR, Gagnon J, Rankinen T, Leon AS, Rao DC, et al The effect of sex, age and race on estimating percentage body fat from body mass index: The Heritage Family Study. International journal of obesity and related metabolic disorders: journal of the International Association for the Study of Obesity. 2002; 26: 789–96. 10.1038/sj.ijo.0802006 12037649

[46] Klesges RC, Klesges LM, Haddock CK, Eck LH. A longitudinal analysis of the impact of dietary intake and physical activity on weight change in adults. Am J Clin Nutr. 1992;55: 818–822. 1550064 10.1093/ajcn/55.4.818

[47] Westerterp KR, Goran MI. Relationship between physical activity related energy expenditure and body composition: a gender difference. International journal of obesity 1997;21:184–188. 9080256 10.1038/sj.ijo.0800385

[48] Lovejoy JC. The influence of sex hormones on obesity across the female life span. J Womens Health. 1998;7:1247–1256. 9929857 10.1089/jwh.1998.7.1247

[49] Slattery ML, McDonald A, Bild DE, Caan BJ, Hilner JE, Jacobs DR, et al Associations of body fat and its distribution with dietary intake, physical activity, alcohol, and smoking in blacks and whites. Am J Clin Nut. 1992; 55: 943–949. 10.1093/ajcn/55.5.943 1570801

[50] Cheskin LJ, Bartlett SJ, Zayas R, Twilley CH, Allison DB, Contoreggi C. Prescription medications: a modifiable contributor to obesity. South Med J. 1999; 92: 898–904. 10498166 10.1097/00007611-199909000-00009

[51] Suter PM, Tremblay A. Is alcohol consumption a risk factor for weight gain and obesity?. Crit Rev Clin Lab Sci. 2005; 42:197–227. 16047538 10.1080/10408360590913542

[52] Hamlin JC, Pauly M, Melnyk S, Pavliv O, Starrett W, Crook TA, et al Dietary intake and plasma levels of choline and betaine in children with autism spectrum disorders. Autism research and treatment 2013. 10.1155/2013/578429 PMC3876775 24396597

[53] Food and Nutrition Board, Institute of Medicine. Dietary Reference Intakes: Thiamin, Riboflavin, Niacin, Vitamin B-6,Vitamin B-12, Pantothenic Acid, Biotin, and Choline National Academy of Sciences. Washington, DC: Food and Nutrition Board, Institute of Medicine National Academic Press; 1998:390–422.

[54] Wu G, Zhang L, Li T, Lopaschuk G, Vance DE, Jacobs RL. Choline deficiency attenuates body weight gain and improves glucose tolerance in ob/ob mice. Journal of obesity, 2012 10.1155/2012/319172 PMC3385711 22778916

[55] Jacobs RL, Zhao Y, Koonen DPY, Sletten T, Su B, Lingrell S, et al Impaired de novo choline synthesis explains why phosphatidylethanolamine N-methyltransferase-deficient mice are protected from diet-induced obesity. J Biol Chem. 2010;285:22403–22413. 10.1074/jbc.M110.108514 20452975 PMC2903412

[56] Raubenheimer PJ, Nyirenda MJ, Walker BR. A choline-deficient diet exacerbates fatty liver but attenuates insulin resistance and glucose intolerance in mice fed a high-fat diet. Diabetes. 2006;55:2015–2020. 16804070 10.2337/db06-0097

[57] Feng J, Xu ZR. Effect of betaine on muscle, liver and serum amino acid composition in finishing swine. J Zhejiang UnivAgric Life Sci. 2001;27:107–110.

[58] Harmeyer J. The physiological role of L-carnitine. Lohman Information. 2002; 27:15–21.

[59] Daily JW, Sachan DS. Choline supplementation alters carnitine homeostasis in humans and guinea pigs. J. Nutr. 1995;125:1938–1944. 7616311 10.1093/jn/125.7.1938

[60] Lawrence BV, Schinckel AP, Adeola O, Cera K. Impact of betaine on pig finishing performance and carcass composition. J Anim Sci. 2002;80: 475–482. 11881932 10.2527/2002.802475x

[61] Huang QC, Xu ZR, Han XY, Li WF. Effect of dietary betaine supplementation on lipogenic enzyme activities and fatty acid synthase mRNA expression in finishing pigs. Anim Feed Sci Technol. 2008;140:365–375.

[62] Xing J, Kang L, Jiang Y. Effect of dietary betaine supplementation on lipogenesis gene expression and CpG methylation of lipoprotein lipase gene in broilers. Mol Biol Rep. 2011;38:1975–1981. 10.1007/s11033-010-0319-4 20845073

[63] Huang QC, Xu ZR, Han XY, Li WF. Changes in hormones, growth factor and lipid metabolism infinishing pigs fed betaine. Livest Sci. 2006;105:78–85.

[64] Najib S, Sanchez-Margalet V. Homocysteine thiolactone inhibits insulin-stimulated DNA and protein synthesis: possible role of mitogen-activated protein kinase (MAPK), glycogen synthase kinase-3 (GSK-3) and p70 S6K phosphorylation. Journal of molecular endocrinology. 2005;34:119–126. 15691882 10.1677/jme.1.01581

[65] Huang QC, Xu ZR, Han XY, Li WF. Effect of betaine on growth hormone pulsatile secretion and serum metabolites in finishing pigs. J Anim Physiol An N. 2007;91: 85–90. 10.1111/j.1439-0396.2006.00644.x 17355337

[66] Apicella JM, Lee EC, Bailey BL, Saenz C, Anderson JM, Craig SAS, et al Betaine supplementation enhances anabolic endocrine and Akt signaling in response to acute bouts of exercise. Eur J Appl Physiol. 2013; 113: 793–802. 10.1007/s00421-012-2492-8 22976217

[67] Slow S, Lever M, Chambers ST, GEORGE PM. Plasma dependent and independent accumulation of betaine in male and female rat tissues. Physiol Res. 2009;58:403 18637704 10.33549/physiolres.931569

[68] Stoll B, Gerok W, Lang F, Häussinger D. Liver cell volume and protein synthesis. Biochem J. 1992;287:217–222. 1329728 10.1042/bj2870217 PMC1133146

[69] Fontaine-Bisson B, Wolever TM, Connelly PW, Corey PN, El-Sohemy A. NF-kappaB—94Ins/Del ATTG polymorphism modifies the association between dietary polyunsaturated fatty acids and HDL-cholesterol in two distinct populations. Atherosclerosis. 2009;204: 465–470. 10.1016/j.atherosclerosis.2008.10.037 19070859

[70] Paradis AM, Godin G, Lemieux S, Pérusse L, Vohl MC. Eating behaviours of non-obese individuals with and without familial history of obesity. Br J Nutr. 2009;101:1103–1109. 10.1017/S0007114508055645 18782460

[71] Eny KM, Wolever TM, Fontaine-Bisson B, El-Sohemy A. Genetic variant in the glucose transporter type 2 is associated with higher intakes of sugars in two distinct populations. Physiol Genomics. 2008;33:355–360. 10.1152/physiolgenomics.00148.2007 18349384

[72] Liu L, Wang PP, Roebothan B, Ryan A, Tucker SC, Colbourne J, et al Assessing the validity of a self-administered food-frequency questionnaire (FFQ) in the adult population of Newfoundland and Labrador, Canada. Nutrition J. 2013;12:49. 23590645 10.1186/1475-2891-12-49 PMC3637508

