# Supplementary figures and images for: Higher Dietary Choline and Betaine Intakes Are Associated with Better Body Composition in the Adult Population of Newfoundland, Canada
Source: PLoS One. 2016 May 11;11(5):e0155403. doi: 10.1371/journal.pone.0155403 (PMC4863971; doi:10.1371/journal.pone.0155403)

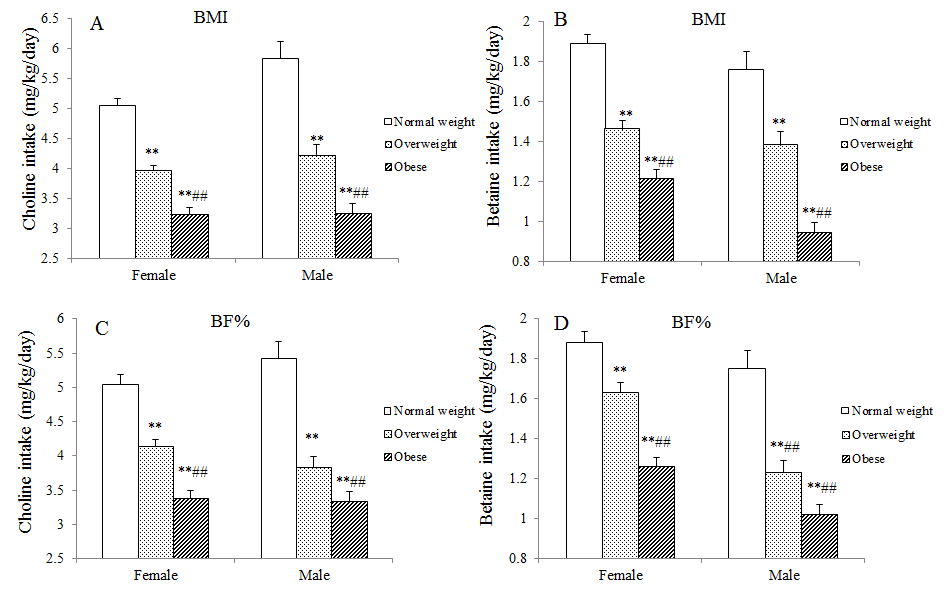

Supplement: S1 Fig — A. variations of dietary choline intake (mg/kg/day) in different obesity status grouped based on BMI criteria; B. variations of dietary betaine intake (mg/kg/day) in different obesity status grouped based on BMI criteria; C. variations of dietary choline intake (mg/kg/day) in different obesity status grouped based on %BF recommended by Bray; D. variations of dietary betaine intake (mg/kg/day) in different obesity status grouped based on %BF recommended by Bray; **p<0.01 compared with normal weight group, ##p<0.01 compared with overweight group. (TIF) [file pone.0155403.s001.tif]

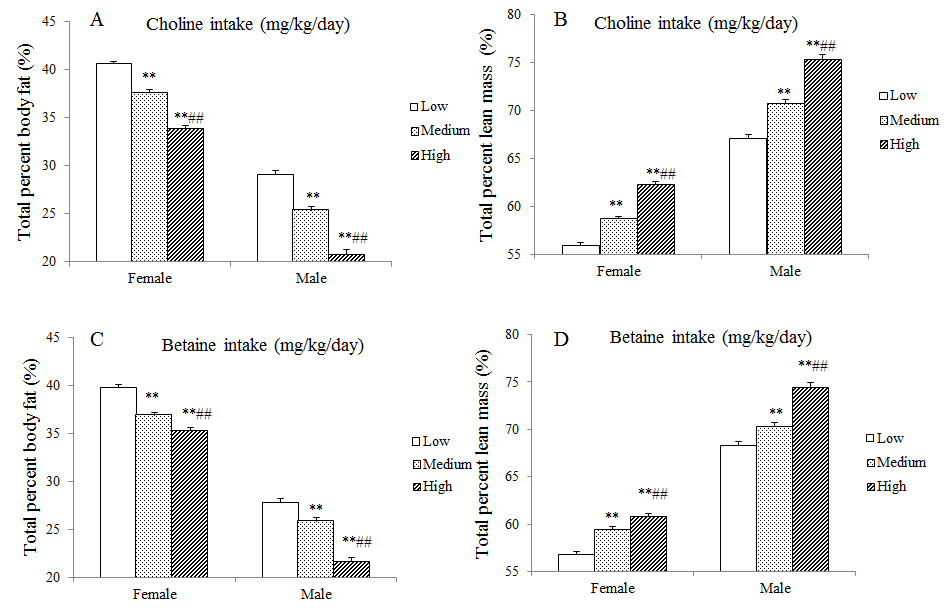

Supplement: S2 Fig — A. variations of total body fat according to dietary choline intake; B. variations of total lean mass according to dietary choline intake; C. variations of total body fat according to dietary betaine intake; D. variations of total lean mass according to dietary betaine intake. **p<0.01 compared with low dietary choline or betaine intakes; ##p< 0.01 compared with medium dietary choline or betaine intakes. (TIF) [file pone.0155403.s002.tif]
