# Supplementary material for: Higher Dietary Choline and Betaine Intakes Are Associated with Better Body Composition in the Adult Population of Newfoundland, Canada
Source: PLoS One. 2016 May 11;11(5):e0155403. doi: 10.1371/journal.pone.0155403 (PMC4863971; doi:10.1371/journal.pone.0155403)
Supplement: S1 Table — (DOC) [file pone.0155403.s003.doc]

**S1 Table. Partial correlations between dietary choline, betaine intakes (mg/kg/day) and body composition variables in females based on menopausal status.*^1^***

|  | Choline (mg/kg/day) | | |  | Betaine (mg/kg/day) | | |
| --- | --- | --- | --- | --- | --- | --- | --- |
|  | Pre-menopausal (n=1394) |  | Post-menopausal (n=838) |  | Pre-menopausal (n=1394) |  | Post-menopausal (n=838) |
|  | r’(p)*^2^* |  | r’(p) *^2^* |  | r’(p) *^2^* |  | r’(p) *^2^* |
| Weight (kg) | -0.441(0.000) |  | -0.479 (0.000) |  | -0.232 (0.000) |  | -0.246 (0.000) |
| BMI (kg/m2) | -0.401(0.000) |  | -0.458 (0.000) |  | -0.231 (0.000) |  | -0.260 (0.000) |
| WC (cm) | -0.412 (0.000) |  | -0.464 (0.000) |  | -0.237 (0.000) |  | -0.236 (0.000) |
| WHR | -0.122 (0.000) |  | -0.171 (0.000) |  | -0.093 (0.000) |  | -0.096 (0.000) |
| Trunk fat (%) | -0.361 (0.000) |  | -0.382 (0.000) |  | -0.228 (0.000) |  | -0.236 (0.000) |
| Android fat (%) | -0.365 (0.000) |  | -0.362 (0.000) |  | -0.229 (0.000) |  | -0.223 (0.000) |
| Gynoid fat (%) | -0.334 (0.000) |  | -0.253 (0.000) |  | -0.168 (0.000) |  | -0.126 (0.000) |
| Total body fat (%) | -0.382 (0.000) |  | -0.375 (0.000) |  | -0.227 (0.000) |  | -0.222 (0.000) |
| Total lean (%) | 0.386 (0.000) |  | 0.373 (0.000) |  | -0.224 (0.000) |  | 0.219 (0.000) |

### *^1^* Partial correlations between dietary choline, betaine intakes (mg/kg/day) and obesity related indexes were controlling for age, total calorie intake, physical activity.

*^2^* r’: partial correlation coefficient.

Statistical significance was set to p<0.05.
