# Supplementary material for: Higher Dietary Choline and Betaine Intakes Are Associated with Better Body Composition in the Adult Population of Newfoundland, Canada
Source: PLoS One. 2016 May 11;11(5):e0155403. doi: 10.1371/journal.pone.0155403 (PMC4863971; doi:10.1371/journal.pone.0155403)
Supplement: S2 Table — (DOC) [file pone.0155403.s004.doc]

**S2 Table. Partial correlations between dietary choline, betaine intakes (mg/kg/day) and body composition variables for Newfoundland population based on medication use.*^1^***

| Choline (mg/kg/day) | Female | | |  | Male | | |
| --- | --- | --- | --- | --- | --- | --- | --- |
|  | Non-medication user (n=922) |  | Medication user (n=1310) |  | Non-medication user (n=525) |  | Medication user (n=297) |
|  | r’(p)*^2^* |  | r’(p) *^2^* |  | r’(p) *^2^* |  | r’(p) *^2^* |
| Weight (kg) | -0.478 (0.000) |  | -0.347 (0.000) |  | -0.372 (0.000) |  | -0.269 (0.000) |
| BMI (kg/m2) | -0.417 (0.000) |  | -0.339 (0.000) |  | -0.373 (0.000) |  | -0.256 (0.000) |
| WC (cm) | -0.432 (0.000) |  | -0.367 (0.000) |  | -0.380 (0.000) |  | -0.268 (0.000) |
| WHR | -0.084 (0.013) |  | -0.156 (0.000) |  | -0.130 (0.003) |  | -0.133 (0.023) |
| Trunk fat (%) | -0.384 (0.000) |  | -0.340 (0.000) |  | -0.360 (0.000) |  | -0.299 (0.000) |
| Android fat (%) | -0.375 (0.000) |  | -0.335 (0.000) |  | -0.375 (0.000) |  | -0.328 (0.000) |
| Gynoid fat (%) | -0.335 (0.000) |  | -0.283 (0.000) |  | -0.310 (0.000) |  | -0.260 (0.000) |
| Total body fat (%) | -0.396 (0.000) |  | -0.346 (0.000) |  | -0.364 (0.000) |  | -0.301 (0.000) |
| Total lean (%) | 0.383 (0.000) |  | 0.336 (0.000) |  | 0.369 (0.000) |  | -0.293 (0.000) |
| Betaine (mg/kg/day) | Female | | |  | Male | | |
|  | Non-medication user (n=922) |  | Medication user (n=1310) |  | Non-medication user (n=525) |  | Medication user (n=297) |
|  | r’(p) *^2^* |  | r’(p) *^2^* |  | r’(p) *^2^* |  | r’(p) *^2^* |
| Weight (kg) | -0.247 (0.000) |  | -0.228 (0.000) |  | -0.191 (0.000) |  | 0.195 (0.000) |
| BMI (kg/m2) | -0.232 (0.000) |  | -0.240 (0.000) |  | -0.193 (0.000) |  | -0.226 (0.000) |
| WC (cm) | -0.219 (0.000) |  | -0.248 (0.000) |  | -0.209 (0.000) |  | -0.184 (0.002) |
| WHR | -0.055 (0.099) |  | -0.120 (0.000) |  | -0.109 (0.014) |  | -0.218 (0.000) |
| Trunk fat (%) | -0.237 (0.000) |  | -0.249 (0.000) |  | -0.263 (0.000) |  | -0.215 (0.000) |
| Android fat (%) | -0.221 (0.000) |  | -0.243 (0.000) |  | -0.253 (0.000) |  | -0.210 (0.000) |
| Gynoid fat (%) | -0.188 (0.000) |  | -0.182 (0.000) |  | -0.138 (0.000) |  | -0.136 (0.021) |
| Total body fat (%) | -0.235 (0.000) |  | -0.246 (0.000) |  | -0.244 (0.000) |  | -0.209 (0.000) |
| Total lean (%) | 0.230 (0.000) |  | 0.234 (0.000) |  | 0.250 (0.000) |  | 0.218 (0.000) |

### *^1^* Partial correlations between dietary choline, betaine intakes (mg/kg/day) and obesity related indexes were controlling for age, total calorie intake, physical activity.

*^2^* r’: partial correlation coefficient. Statistical significance was set to p<0.05.
