# Supplementary material for: Higher Dietary Choline and Betaine Intakes Are Associated with Better Body Composition in the Adult Population of Newfoundland, Canada
Source: PLoS One. 2016 May 11;11(5):e0155403. doi: 10.1371/journal.pone.0155403 (PMC4863971; doi:10.1371/journal.pone.0155403)
Supplement: S3 Table — (DOC) [file pone.0155403.s005.doc]

**S3 Table. Partial correlations between dietary choline, betaine intakes (mg/kg/day) and body composition variables for Newfoundland population based on smoking status.*^1^***

| Choline (mg/kg/day) | Female | | |  | Male | | |
| --- | --- | --- | --- | --- | --- | --- | --- |
|  | Non-smoking (n=1997) |  | Smoking (n=235) |  | Non- smoking (n=729) |  | Smoking  (n=93) |
|  | r’(p)*^2^* |  | r’(p) *^2^* |  | r’(p) *^2^* |  | r’(p) *^2^* |
| Weight (kg) | -0.491 (0.000) |  | -0.416 (0.000) |  | -0.289 (0.000) |  | -0.481 (0.000) |
| BMI (kg/m2) | -0.449 (0.000) |  | -0.386 (0.000) |  | -0.273 (0.000) |  | -0.486 (0.000) |
| WC (cm) | -0.447 (0.000) |  | -0.493 (0.000) |  | -0.283 (0.000) |  | -0.521 (0.000) |
| WHR | -0.136 (0.000) |  | -0.217 (0.000) |  | -0.126 (0.000) |  | -0.220 (0.038) |
| Trunk fat (%) | -0.385 (0.000) |  | -0.344 (0.000) |  | -0.355 (0.000) |  | -0.267 (0.011) |
| Android fat (%) | -0.375 (0.000) |  | -0.334 (0.000) |  | -0.347 (0.000) |  | -0.336 (0.001) |
| Gynoid fat (%) | -0.325 (0.000) |  | -0.291 (0.000) |  | -0.297 (0.000) |  | -0.322 (0.002) |
| Total body fat (%) | -0.398 (0.000) |  | -0.350 (0.000) |  | -0.332 (0.000) |  | -0.302 (0.004) |
| Total lean (%) | 0.387 (0.000) |  | 0.342 (0.000) |  | 0.339 (0.000) |  | 0.286 (0.007) |
| Betaine (mg/kg/day) | Female | | |  | Male | | |
|  | Non-smoking (n=1997) |  | Smoking (n=235) |  | Non- smoking (n=729) |  | Smoking  (n=93) |
|  | r’(p) *^2^* |  | r’(p) *^2^* |  | r’(p) *^2^* |  | r’(p) *^2^* |
| Weight (kg) | -0.248 (0.000) |  | -0.256 (0.000) |  | -0.220 (0.000) |  | -0.139 (0.195) |
| BMI (kg/m2) | -0.249 (0.000) |  | -0.260 (0.000) |  | -0.235 (0.000) |  | -0.184 (0.087) |
| WC (cm) | -0.238 (0.000) |  | -0.299 (0.000) |  | -0.208 (0.000) |  | -0.188 (0.079) |
| WHR | -0.087 (0.000) |  | -0.136 (0.000) |  | -0.166 (0.000) |  | -0.197 (0.065) |
| Trunk fat (%) | -0.227 (0.000) |  | -0.276 (0.000) |  | -0.276 (0.000) |  | -0.151 (0.159) |
| Android fat (%) | -0.335 (0.000) |  | -0.263 (0.000) |  | -0.267 (0.000) |  | -0.148 (0.168) |
| Gynoid fat (%) | -0.160 (0.000) |  | -0.243 (0.000) |  | -0.164 (0.000) |  | -0.119 (0.271) |
| Total body fat (%) | -0.229 (0.000) |  | -0.279 (0.000) |  | -0.254 (0.000) |  | -0.188 (0.079) |
| Total lean (%) | 0.221 (0.000) |  | 0.269 (0.000) |  | 0.266 (0.000) |  | 0.181 (0.091) |

### *^1^* Partial correlations between dietary choline, betaine intakes (mg/kg/day) and obesity related indexes were controlling for age, total calorie intake, physical activity.

*^2^* r’: partial correlation coefficient. Statistical significance was set to p<0.05.
