# Supplementary material for: Higher Dietary Choline and Betaine Intakes Are Associated with Better Body Composition in the Adult Population of Newfoundland, Canada
Source: PLoS One. 2016 May 11;11(5):e0155403. doi: 10.1371/journal.pone.0155403 (PMC4863971; doi:10.1371/journal.pone.0155403)
Supplement: S4 Table — (DOC) [file pone.0155403.s006.doc]

**S4 Table. Partial correlations between dietary choline, betaine intakes (mg/kg/day) and body composition variables for Newfoundland population based on alcohol status.*^1^***

| Choline (mg/kg/day) | Female | | |  | Male | | |
| --- | --- | --- | --- | --- | --- | --- | --- |
|  | Non-alcohol (n=569) |  | Alcohol (n=1663) |  | Non-alcohol (n=137) |  | Alcohol  (n=685) |
|  | r’(p)*^2^* |  | r’(p) *^2^* |  | r’(p) *^2^* |  | r’(p) *^2^* |
| Weight (kg) | -0.511 (0.000) |  | -0.464 (0.000) |  | -0.363 (0.000) |  | -0.306 (0.000) |
| BMI (kg/m2) | -0.480 (0.000) |  | -0.421 (0.000) |  | -0.295 (0.000) |  | -0.320 (0.000) |
| WC (cm) | -0.502 (0.000) |  | -0.429 (0.000) |  | -0.270 (0.002) |  | -0.341 (0.000) |
| WHR | -0.222 (0.000) |  | -0.114 (0.000) |  | -0.202 (0.023) |  | -0.148 (0.000) |
| Trunk fat (%) | -0.398 (0.000) |  | -0.370 (0.000) |  | -0.309 (0.000) |  | -0.346 (0.000) |
| Android fat (%) | -0.420 (0.000) |  | -0.361 (0.000) |  | -0.341 (0.000) |  | -0.364 (0.000) |
| Gynoid fat (%) | -0.313 (0.000) |  | -0.316 (0.000) |  | -0.277 (0.000) |  | -0.304 (0.000) |
| Total body fat (%) | -0.403 (0.000) |  | -0.382 (0.000) |  | -0.302 (0.000) |  | -0.346 (0.000) |
| Total lean (%) | 0.396 (0.000) |  | 0.371 (0.000) |  | 0.291 (0.000) |  | 0.349 (0.000) |
| Betaine (mg/kg/day) | Female | | |  | Male | | |
|  | Non-alcohol (n=569) |  | Alcohol (n=1663) |  | Non-alcohol (n=137) |  | Alcohol  (n=685) |
|  | r’(p) *^2^* |  | r’(p) *^2^* |  | r’(p) *^2^* |  | r’(p) *^2^* |
| Weight (kg) | -0.269 (0.000) |  | -0.234 (0.000) |  | -0.189 (0.000) |  | -0.208 (0.000) |
| BMI (kg/m2) | -0.316 (0.000) |  | -0.219 (0.000) |  | -0.196 (0.028) |  | -0.234 (0.000) |
| WC (cm) | -0.295 (0.000) |  | -0.221 (0.000) |  | -0.127 (0.157) |  | -0.231 (0.000) |
| WHR | -0.123 (0.000) |  | -0.082 (0.000) |  | -0.185 (0.038) |  | -0.171 (0.000) |
| Trunk fat (%) | -0.291 (0.000) |  | -0.216 (0.000) |  | -0.166 (0.064) |  | -0.275 (0.000) |
| Android fat (%) | -0.269 (0.000) |  | -0.215 (0.000) |  | -0.153 (0.087) |  | -0.271 (0.000) |
| Gynoid fat (%) | -0.194 (0.000) |  | -0.154 (0.000) |  | -0.117 (0.192) |  | -0.158 (0.000) |
| Total body fat (%) | -0.291 (0.000) |  | -0.210 (0.000) |  | -0.185 (0.038) |  | -0.252 (0.000) |
| Total lean (%) | 0.279 (0.000) |  | 0.203 (0.000) |  | 0.183 (0.041) |  | 0.262 (0.000) |

### *^1^* Partial correlations between dietary choline, betaine intakes (mg/kg/day) and obesity related indexes were controlling for age, total calorie intake, physical activity.

*^2^* r’: partial correlation coefficient. Statistical significance was set to p<0.05.
