# Supplementary material for: Higher Dietary Choline and Betaine Intakes Are Associated with Better Body Composition in the Adult Population of Newfoundland, Canada
Source: PLoS One. 2016 May 11;11(5):e0155403. doi: 10.1371/journal.pone.0155403 (PMC4863971; doi:10.1371/journal.pone.0155403)
Supplement: S5 Table — (DOC) [file pone.0155403.s007.doc]

**S5 Table. Correlations between dietary choline and betaine intakes (mg/day) with body composition*^1^***

| Choline intake  (mg/day) | Female (n=2232) | | |  | Male (n=822) | | |
| --- | --- | --- | --- | --- | --- | --- | --- |
|  | r^2^ | r’^2^ | *p* for r’^3^ |  | r^2^ | r’^2^ | *p* for r’^3^ |
| Weight (kg) | -0.03 | -0.02 | 0.493 |  | -0.03 | -0.07 | 0.130 |
| BMI (kg/m^2^) | -0.04 | -0.02 | 0.549 |  | -0.12 | -0.04 | 0.529 |
| WC (cm) | -0.10 | -0.07 | 0.002 |  | -0.19 | -0.01 | 0.834 |
| WHR | -0.01 | -0.01 | 0.898 |  | -0.10 | -0.04 | 0.294 |
| Trunk fat (%) | -0.12 | -0.04 | 0.068 |  | -0.28 | -0.08 | 0.017 |
| Android fat (%) | -0.12 | -0.06 | 0.005 |  | -0.30 | -0.13 | 0.000 |
| Gynoid fat (%) | -0.13 | -0.07 | 0.002 |  | -0.21 | -0.11 | 0.002 |
| Total body fat (%) | -0.12 | -0.05 | 0.049 |  | -0.26 | -0.09 | 0.020 |
| Total lean (%) | 0.12 | 0.05 | 0.048 |  | 0.26 | 0.09 | 0.009 |
| Betaine intake | Female (n=2232) | | |  | Male (n=822) | | |
| (mg/day) | r^2^ | r’^2^ | *p* for r’^3^ |  | r^2^ | r’^2^ | *p* for r’^3^ |
| Weight (kg) | -0.03 | -0.02 | 0.390 |  | -0.04 | -0.02 | 0.888 |
| BMI (kg/m^2^) | -0.06 | -0.04 | 0.057 |  | -0.12 | -0.04 | 0.222 |
| WC (cm) | -0.08 | -0.06 | 0.011 |  | -0.13 | -0.03 | 0.353 |
| WHR | -0.02 | -0.03 | 0.228 |  | -0.13 | -0.07 | 0.045 |
| Trunk fat (%) | -0.12 | -0.07 | 0.002 |  | -0.23 | -0.12 | 0.001 |
| Android fat (%) | -0.11 | -0.08 | 0.001 |  | -0.22 | -0.12 | 0.001 |
| Gynoid fat (%) | -0.10 | -0.04 | 0.059 |  | -0.13 | -0.04 | 0.225 |
| Total body fat (%) | -0.11 | -0.06 | 0.005 |  | -0.21 | -0.10 | 0.005 |
| Total lean (%) | 0.11 | 0.06 | 0.011 |  | 0.22 | 0.11 | 0.001 |

*^1^*Partial correlations between dietary choline, betaine (mg/day) intakes and obesity related indexes were controlling for age, total calorie intake, physical activity level. BMI, body mass index; WC, waist circumference; WHR, waist-to-hip ratio.

*^2^*r: correlation coefficient; r’: partial correlation coefficient.

*^3^*Statistical significance was set to p<0.05.
